# Psychological primitives can make sense of biopsychosocial factor complexity in psychopathology

**DOI:** 10.1186/s12916-019-1435-1

**Published:** 2019-10-18

**Authors:** Joseph C. Franklin

**Affiliations:** Department of Psychology, Florida State University, 1107 W Call St, Tallahassee, FL 32304 USA

**Keywords:** Complexity, Psychological primitives, Psychopathology, Suicide, Indeterminacy

## Abstract

**Background:**

Many agree that the biopsychosocial contributions to psychopathology are complex, yet it is unclear how we can make sense of this complexity. One approach is to reduce this complexity to a few necessary and sufficient biopsychosocial factors; although this approach is easy to understand, it has little explanatory power. Another approach is to fully embrace complexity, proposing that each instance of psychopathology is caused by a partially unique set of biopsychosocial factors; this approach has high explanatory power, but is impossible to comprehend. Due to deficits in either explanatory power or comprehensibility, both approaches limit our ability to make substantial advances in understanding, predicting, and preventing psychopathology. Thus, how can we make sense of biopsychosocial factor complexity?

**Main text:**

There is a third possible approach that can resolve this dilemma, with high explanatory power and high comprehensibility. This approach involves understanding, predicting, and preventing psychopathology in terms of a small set of psychological primitives rather than biopsychosocial factors. Psychological primitives are the fundamental and irreducible elements of the mind, mediating all biopsychosocial factor influences on psychopathology. All psychological phenomena emerge from these primitives. Over the past decade, this approach has been successfully applied within basic psychological science, most notably affective science. It explains the sum of the evidence in affective science and has generated several novel research directions. This approach is equally applicable to psychopathology. The primitive-based approach does not eliminate the role of biopsychosocial factors, but rather recasts them as indeterminate causal influences on psychological primitives. In doing so, it reframes research away from factor-based questions (e.g., which situations cause suicide?) and toward primitive-based questions (e.g., how are suicidality concepts formed, altered, activated, and implemented?). This is a valuable shift because factor-based questions have indeterminate answers (e.g., infinite situations could cause suicide) whereas primitive-based questions have determinate answers (e.g., there are specific processes that undergird all concepts).

**Conclusion:**

The primitive-based approach accounts for biopsychosocial complexity, ties clinical science more directly to basic psychological science, and could facilitate progress in understanding, predicting, and preventing psychopathology.

## Background

In recent years, many researchers have converged on the view that biopsychosocial factor contributions to psychopathology are complex. Some have proposed that this complexity necessitates complicated models of psychopathology that may include tens, hundreds, or even thousands of factors; although this approach has high explanatory power, it is too complex for humans to comprehend. As a result, it precludes the development of useful theories and viable intervention targets. Others have proposed that complex biopsychosocial factor contributions to psychopathology can be reduced to a handful of factors (sometimes posited as hierarchical or latent factors); although this approach has high comprehensibility, it has low explanatory power. Consequently, this approach also struggles to produce accurate theories or effective intervention targets. Neither the simple approach nor the complex approach seem to permit major advances in understanding or preventing psychopathology, yet they have often appeared as the only two possible approaches. A resolution to this simple–complex dilemma involves a third approach – understanding psychopathology in terms of psychological primitives rather than biopsychosocial factors. This move permits biopsychosocial complexity while providing a nomothetic explanation simple enough for humans to apply to advance the understanding, prediction, and prevention of psychopathology; this approach is described herein.

### Simple, complicated, and complex

To understand the profound implications of complexity for psychopathology, it is necessary to understand how complex systems differ from simple and complicated systems. Complexity clearly challenges most traditional views of psychopathology, which propose that simple sets of biopsychosocial factors (e.g., 1–10 factors) are necessary and sufficient to account for specific forms of psychopathology. However, the complex view also challenges the perspective that biopsychosocial factor contributions are complicated. ‘Complicated’ is often confused with ‘complex’, but there are many important differences between these two types of systems [1, 2]. Complicated systems may include thousands of factors that fit together in a mechanistic, linear, and determinate fashion to produce a particular phenomenon. For example, an airplane is complicated. It has thousands of parts, but those parts must be combined in a specific way for the airplane to fly. In contrast, complex systems contain a large number of factors, but those factors fit together in a probabilistic, nonlinear, and indeterminate fashion. For example, climates, economies, ant colonies, and the immune system are complex systems – they possess many parts, and there are many (and perhaps infinite) ways for those parts to fit together to produce a given outcome. For instance, there are infinite ways for climatological factors to combine to produce a sea level rise. A comprehensive summary of complex adaptive systems is beyond the scope of this review [3, 4]; given space limitations, this review will focus on how just one general feature of complex systems – indeterminacy – is sufficient to greatly limit the ability to understand, predict, and prevent psychopathology in terms of biopsychosocial factors.

Indeterminacy is a core feature of complex systems [5–8]. It is sometimes referred to as degeneracy or equifinality, but due to some confusion over these latter two terms [6], this review will refer to this property as indeterminacy. Indeterminacy can be illustrated with math equations such as X + Y = 1. This simple equation has infinite correct solutions, which means that the values for X and Y cannot be specifically determined (i.e., indeterminate); however, this does not imply that the solutions to this equation are random or ‘anything goes’. Infinity refers to an uncountable limit, not randomness or a lack of order. Indeed, there are also infinite values for X and Y that would make this equation false.

If biopsychosocial factor contributions to psychopathology are complex, as much converging evidence indicates, then these factor contributions are indeterminate – this means that infinite biopsychosocial factors and factor combinations may contribute to a given form of psychopathology. Once again, this does not mean that biopsychosocial factor contributions to psychopathology are random; however, it does mean that trying to identify factor contributions to psychopathologies is like trying to identify the specific numerical combinations that make X + Y = 1 true. It is a Sisyphean task – to account for indeterminate biopsychosocial factor contributions, accurate nomothetic theories and prediction algorithms would need be to be infinitely large, and effective nomothetic interventions would need to target an infinite set of biopsychosocial factors. But humans cannot comprehend infinitely large theories, and infinitely large prediction algorithms and intervention targets are not possible. This creates a dilemma – simple and complicated approaches have limited explanatory power because they leave out many relevant factors and factor combinations; however, truly complex approaches, despite having a high degree of explanatory power, do not permit comprehensible theories, practical prediction algorithms, or feasible intervention approaches. To resolve this dilemma, we must consider psychopathology in terms of something other than biopsychosocial factors.

### Struggles to make sense of complex biopsychosocial contributions to suicidality

To illustrate the simple–complex dilemma and how it might be resolved, this article will continually draw on suicidality^1^ as an example. Suicidality is a particularly apt example because it is transdiagnostic and intense debates have recently risen about how to best make sense of complex biopsychosocial contributions to suicidality.

Several lines of evidence indicate that biopsychosocial contributions to suicidality are complex rather than complicated or simple. Due to space limitations, only a few of these lines are noted here to illustrate this point. For example, meta-analyses show that no known factor or small combination of factors – out of hundreds tested – predicts suicidality much better than random guessing [9]. Similarly, no known factor or small set of factors produces accurate cross-sectional classification of suicide ideators and suicide attempters [10, 11]. Complicated machine learning models that include tens or even hundreds of factors can accurately predict and classify suicidality [10, 12–14]; however, even these complicated models do not produce perfect accuracy and they struggle to replicate in new samples. Moreover, consistent with indeterminacy, algorithms with very different factors and factor combinations can produce accurate classification and prediction of suicidality, even in the same sample [10, 12–14]. This general pattern is indicative of complexity. One implication of this complexity is that suicidality interventions that target a simple (e.g., serotonin levels) or complicated set of factors (e.g., multiple facets of emotion regulation, distress tolerance, interpersonal skills) should have limited success because each target has limited relevance to suicidality. Consistent with this implication, a recent meta-analysis of over 300 randomized controlled trials with suicidality as an outcome found that all known interventions produce, at best, small reductions in suicidality (~ 8–15%) [15].

Reasoning from these and similar lines of evidence, some researchers have concluded that suicidality theory, prediction, and treatment must reflect biopsychosocial complexity, namely that suicidality cannot be reduced to specific factors [14, 16]. Other researchers have proposed that complex approaches are not parsimonious and that simple sets of biopsychosocial factors (often proposed as hierarchical or latent factors) can account for complex contributions to suicidality (e.g., [17, 18]). Despite many intriguing theories and decades of research, however, there is no evidence that any simple model comes close to accounting for broad patterns of evidence on suicidality (e.g., [9–15]). Even complicated models optimized with machine learning cannot accomplish this, so it is no surprise that more simple models fall short. This deficit is perhaps most obvious with suicidality prediction [9, 12–14]; however, deficits are similar for cross-sectional classification of suicidality (e.g., [10, 11]) and the identification of effective intervention targets [15]. Proponents of the complex approach have accordingly pointed out that simple models are not the most parsimonious models of suicidality. Parsimony does not propose that simpler models are necessarily better; it proposes that, among models with identical explanatory power, the simplest is best. Explanatory power refers to the ability to explain all evidence on a phenomenon, not just narrow lines of evidence. Because the complex approach is currently the simplest way to explain the sum of the evidence on suicidality, it is currently the most parsimonious explanation for suicidality.

This fact is unfortunate because, as proponents of simple models rightly indicate, the complex approach has low comprehensibility – we cannot make sense of infinite potential biopsychosocial contributions to suicidality. We accordingly cannot use the complex approach to further understand, predict, or prevent suicidality. To advance suicidality science, we must develop a theory of suicidality that has both high comprehensibility and high explanatory power. Due to indeterminacy, however, such a theory is not possible at the biopsychosocial level. Therefore, how can we develop such theories of suicidality and other forms of psychopathology?

### Psychological primitives account for biopsychosocial complexity

Psychological primitives offer a solution to this simple–complex dilemma (Table 1). This approach is analogous to understanding X + Y = 1 in terms of X and Y themselves rather than the infinite specific values that they could represent.

**Table 1:** Overview of four general approaches to explaining psychopathology. *Notes.* Traditionally, research has focused on the simple biopsychosocial approach, with more recent research focusing on complicated and complex biopsychosocial approaches. The psychological primitive approach permits biopsychosocial complexity while maintaining high comprehensibility. It also ties psychopathology research directly to basic psychological science because non-pathological phenomena emerge from these same psychological primitives

	Claims	Objective	Comprehensibility	Explanatory power
**Simple biopsychosocial**	A small set of necessary and sufficient factors fully explains a given psychopathological phenomenon	Identify the small set of necessary and sufficient factors that fully explains a given psychopathological phenomenon	High	Low
**Complicated biopsychosocial**	A complicated set of necessary and sufficient factors fully explains a given psychopathological phenomenon	Identify the complicated set of necessary and sufficient factors that fully explains a given psychopathological phenomenon	Low-to-moderate	Low-to-moderate
**Complex biopsychosocial**	Factor associations with psychopathology are indeterminate; there are no nomothetic factor-based explanations for psychopathology, only idiographic explanations	Identify the necessary and sufficient set of factors that explains a given instance of a psychopathological phenomenon; these factors will vary across instances such that a viable nomothetic factor-based explanation is not possible	Low	High
**Psychological primitives**	Because factor associations are indeterminate, psychopathology is best explained in terms of a small set of psychological primitives; factors can influence the primitives from which psychopathological phenomena emerge	Understand the basic science of psychological primitives (e.g., concepts); apply this to advance the understanding, prediction, and prevention of psychopathology (e.g., an intervention that disrupts the suicidality concept)	High	High

### What are psychological primitives?

The word primitive comes from the Latin *primus*, which means first or original. Primitives of any kind, not just of the psychological kind, refer to the fundamental and irreducible entities from which all others of that kind originate [19–25]. For example, chemical elements are the chemical primitives from which all molecules originate; they are fundamental (i.e., do not rely on anything else chemical to exist) and irreducible (i.e., cannot be reduced to any other chemicals) at the chemical level. Psychological primitives are the fundamental (i.e., do not rely on anything else psychological to exist) and irreducible (i.e., cannot be reduced to anything else psychological) psychological entities from which all psychological phenomena emerge [20–25].

**Fig. 1:**
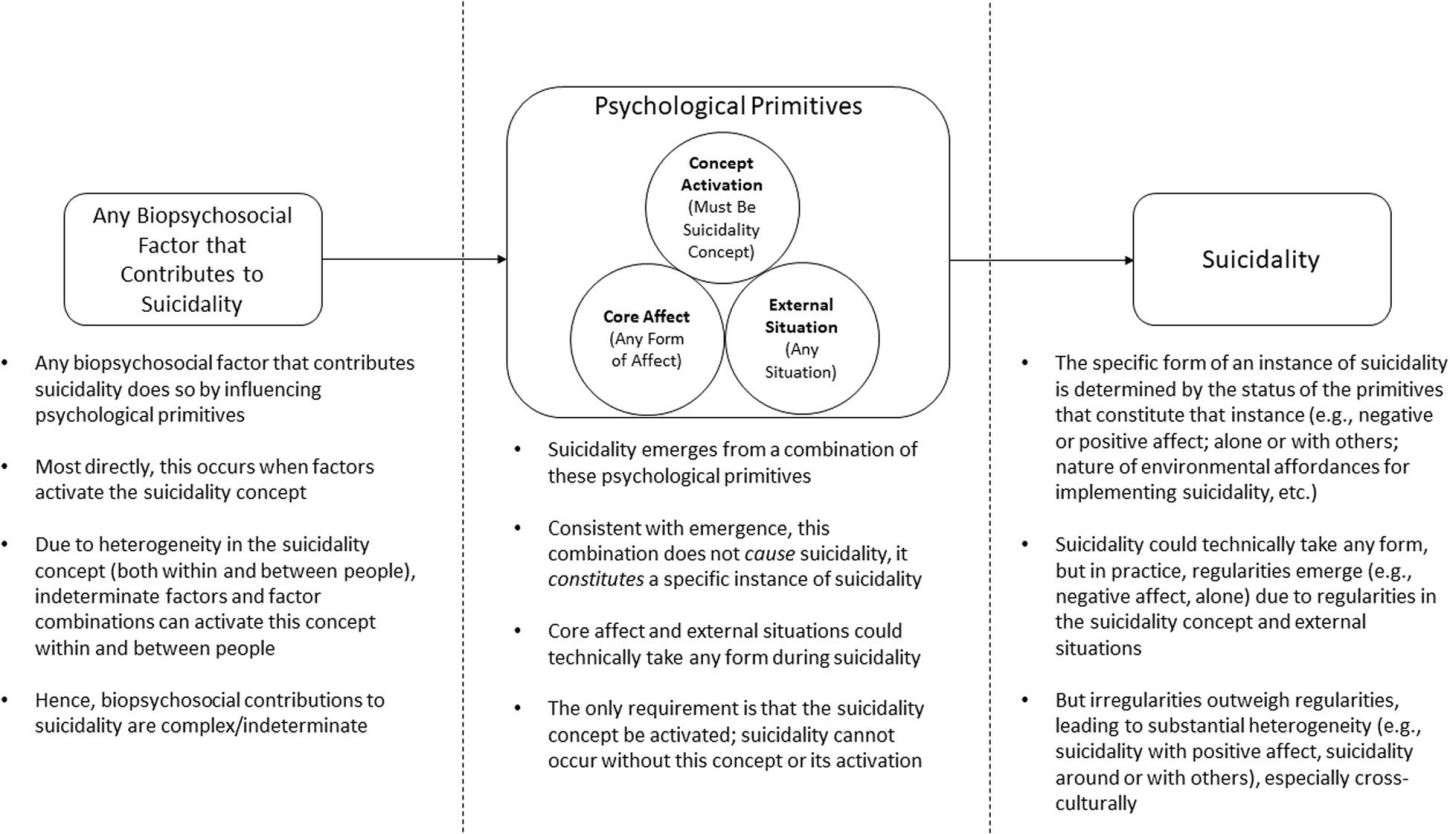
Example of how suicidality could be explained from a psychological primitive perspective. Suicidality emerges from three psychological primitives – conceptual knowledge, interoception (i.e., core affect), and exteroception (i.e., external situation). That is, suicidality occurs when someone makes sense of their ongoing internal and external stimuli as suicidality based on their conceptual knowledge about suicidality. For suicidality to emerge, the suicidality concept must be activated. Technically, core affect and external situations could take any form during activation of the suicidality concept. In practice, however, regularities in these two other primitives emerge due to regularities in the suicidality concept and external situations. Psychological primitives mediate the association between biopsychosocial factors and suicidality. This approach accounts for both heterogeneity in biopsychosocial contributions to suicidality (i.e., complex/indeterminate biopsychosocial contributions to suicidality) and heterogeneity in suicidality itself (i.e., indeterminate features of suicidality, variation in suicidality across cultures). This approach suggests that research should focus on understanding how suicidality concepts are formed, altered, disrupted, activated, and implemented

All (non-primitive) psychological phenomena can be understood in terms of emergence from these primitives; this is analogous to how all non-primitive chemical phenomena (i.e., molecules) can be understood in terms of emergence from a combination of chemical primitives (i.e., chemical elements). Because all psychological phenomena emerge directly from these primitives, psychological primitives mediate the complex effects of biopsychosocial factors on psychological phenomena (Fig. 1); this is analogous to how chemical elements mediate the complex effects of physical, chemical, and biological factors on molecules. Because of this mediation, biopsychosocial factor contributions to a given psychological phenomenon are best understood in terms of how those factors influence the primitives that give rise to that phenomenon (Fig. 1). As a result, this approach provides a comprehensible explanation for each psychological phenomenon (i.e., explanations are in terms of a small set of psychological primitives) while accounting for a complex biopsychosocial factor contribution to each phenomenon (i.e., indeterminate biopsychosocial factors can influence each primitive).

The identification of psychological primitives was a major objective at the outset of psychological science [26–28], though early researchers typically used terms such as elemental processes rather than psychological primitives (e.g., [27]). This focus was lost with the rise of behaviorism and subsequent schools of psychology. Nonetheless, a few researchers continued to search for psychological primitives. For example, some posited that emotions such as fear were psychological primitives. However, this position did not hold up to scrutiny [24] – evidence indicated that these emotions are neither psychologically fundamental nor psychologically irreducible. Nevertheless, over the past few decades, many basic psychological science researchers have obtained support for proposed psychological primitives. Most prominently, an approach called psychological constructionism (specifically, the Conceptual ACT Theory) [20–23] has identified as least three psychological primitives, namely interoception (i.e., internal stimuli, often referred to as ‘core affect’ because it is psychologically represented as affect), exteroception (i.e., external stimuli), and conceptual knowledge (i.e., mental categories composed of heterogenous and dynamic exemplars based on prior experiences). Several broad lines of evidence support these three as psychological primitives (e.g., [29–34]).

From this psychological constructionist perspective, all psychological phenomena emerge from (i.e., are constructed from) these primitives. Specifically, a psychological phenomenon occurs when someone makes sense of their ongoing internal (i.e., core affect) and external (i.e., external situation) stimuli in terms of their conceptual knowledge (i.e., prior experience). Among these primitives, conceptual knowledge plays the crucial role of organizing, identifying, and inferring information about sets of stimuli (i.e., determining whether something is food, dangerous, joyful, metal, etc.). To illustrate this novel approach, two examples are outlined below.

### Psychological primitives applied to affective science

Primitive-based approaches have been most prominently applied in affective science (but see also [20, 23, 29, 33, 35]). Over the past 15 years, this approach has become increasingly popular in affective science because of its explanatory power, comprehensibility, and ability to provide fruitful new research directions. It represents a case study of the benefits of adopting a primitive-based approach; its application to psychopathology may generate similar benefits.

As in most areas of psychological science, many traditional theories of emotion are simple, reducing emotions to a small set of biopsychosocial factors (e.g., [22, 37]). For example, popular theories reduce emotion to specific brain areas (e.g., fear is determined by amygdala activity), psychological qualities (e.g., fear occurs when arousal is high and valence is negative), and/or social situations (e.g., fear occurs in response to an acute threat). Unfortunately, such theories have low explanatory power because biopsychosocial factor contributions to emotions have been shown to be complex and indeterminate. For example, no brain area is specifically associated with fear; fear can occur during high or low arousal as well as during negative or positive valence; and fear can occur in response to many different types of situations, and acute threat can prompt emotions other than fear [22, 30–34, 36, 37]. Within samples, complicated machine learning models can accurately classify emotions. However, consistent with indeterminacy, even these models are imperfect, they struggle to replicate in new samples, and very different algorithms can produce similarly accurate classification (e.g., [34, 38]). Like the data on suicidality described above, the sum of the evidence indicates biopsychosocial factor contributions to emotions are complex – too complex for humans to craft comprehensible theories at the biopsychosocial level.

Psychological primitive approaches such as psychological constructionism provide a way forward by accounting for biopsychosocial complexity while maintaining comprehensibility. For example, according to psychological constructionism, fear occurs when someone makes meaning of their ongoing internal stimuli (i.e., core affect) and external stimuli (i.e., external situation) as fear based on their conceptual knowledge (i.e., prior experiences that have been categorized as fear) [20–22]. Matching the sum of the evidence, this account allows fear to be associated with any form of core affect (i.e., high/low arousal, positive/negative affect), any situation (i.e., threat or no threat), and any type of brain activity (e.g., high or low amygdala activity).

From this perspective, fear has only one necessary component – activation of conceptual knowledge about fear. Biopsychosocial factors are only relevant to fear insomuch as they are relevant to a given person’s fear concept. However, as with all concepts [39], the fear concept is not monolithic – it is highly heterogeneous within people, across people, and especially across cultures. In other words, the fear concept has indeterminate features. Once again, this indeterminacy does not imply randomness. Concepts are powerfully influenced by sociodevelopmental processes (e.g., [40]), meaning that conceptual regularities develop within individuals and cultures. Therefore, some regularities should develop in the affective, situational, and biological correlates of phenomena like fear – especially within individuals and cultures – yet these regularities are typically outweighed by irregularities.

This approach refocuses affective science away from factor-based questions (e.g., which situations cause fear?) and toward primitive-based questions (e.g., how are fear concepts formed, altered, activated, and implemented?). This shift is valuable because factor-based questions have indeterminate answers (e.g., infinite situations can cause fear) whereas primitive-based questions have determinate answers (e.g., there are specific processes through which concepts are formed, altered, activated, and implemented (e.g., [35, 39, 40]). The primitive-based approach has generated several novel hypotheses about emotions that are strongly supported by empirical evidence (e.g., [29–34, 37, 38, 41–47]).

### Applying psychological primitives to suicidality

In terms of psychological primitives, suicidality would be explained as occurring when someone makes sense of their ongoing internal stimuli (i.e., core affect) and external stimuli (i.e., external situation) as suicidality based on their conceptual knowledge (i.e., prior experiences that they, or others in communication with them, categorized as suicide related). Matching the sum of the evidence described above, this account permits complex biopsychosocial contributions to suicidality. Unlike existing theories, it does not propose that suicidality must be associated with any particular biological, psychological, or social factor – it posits that biopsychosocial factors will be relevant to suicidality inasmuch as they are relevant to one’s suicidality concept.

As with all concepts, the suicidality concept is likely to be composed of heterogenous exemplars within-person (e.g., exemplars of depressed/lonely suicide, self-sacrificial suicide, vengeful suicide, assisted suicide, murder-suicide, ritual suicide, mass suicide, etc.) and likely to vary substantially across cultural groups (e.g., predominance of honor-related suicides in certain cultures). The heterogeneity of the suicidality concept means that a wide array of biopsychosocial factors can activate the suicidality concept, and what activates this concept for one person may not activate it for another. Moreover, what activates the suicidality concept for a given person at one point in time may not be the same as what activates it for them at another point in time. In other words, conceptual heterogeneity translates into complex biopsychosocial contributions to suicidality both within and across people. However, again, this does not mean that biopsychosocial contributions to suicidality are random. Sociodevelopmental processes generate regularities in concepts, which generates regularities in biopsychosocial factors that activate these concepts. Nevertheless, these regularities are outweighed by irregularities, resulting in indeterminate biopsychosocial contributions to suicidality.

The primitive-based approach reframes many of the fundamental questions in suicidality research away from factor-based questions (e.g., what situations cause suicidality?) and toward primitive-based questions (e.g., how are suicidality concepts formed, altered, activated, and implemented?). As in affective science, this shift is valuable because factor-based questions have indeterminate answers (e.g., potentially infinite situations could cause suicidality), whereas primitive-based questions have determinate answers (e.g., all concepts are formed, altered, activated, and implemented via specific processes). The primitive-based approach accordingly facilitates linear scientific progress. It also generates several novel and highly testable hypotheses for suicide science, a few of which are noted below for illustration purposes.

1. It is impossible to experience suicidality in the absence of a suicidality concept. Without this concept, one cannot make sense of ongoing internal and external stimuli as suicidality. This provides a new direction for suicidality interventions, namely the disruption or elimination of the suicidality concept. One way to disrupt this concept would be to disrupt its reconsolidation with propranolol, an approach that has been successfully applied to disrupt traumatic memories [48].
2. The development of suicidality should coincide with the development of a suicidality concept. Indirect evidence supports this hypothesis as suicidality rates jump in early adolescence [49], when suicidality concepts tend to mature [50].
3. Suicidality is not necessarily associated with extreme negative affect. There should be heterogenous affective associations with suicidality, including some instances where suicidality occurs in the context of positive affect. Indirect evidence supports this hypothesis [51, 52] yet, surprisingly, studies have rarely directly examined affect during suicidality.
4. Given the right situation, suicidal behavior can occur suddenly in almost anyone. For example, in a situation where the choice is between killing oneself and killing several friends (e.g., jumping on a live grenade), many people may make the sudden choice to kill themselves. Although more gradual processes and psychopathology may commonly contribute to suicidal behavior, they are not necessary for suicidal behavior. From a primitive-based perspective, all that is necessary for suicidal behavior is for an individual to conceptualize suicidal behavior as having a valued function in a given situation (i.e., attainment of something desired, avoidance of something undesired). Biopsychosocial factors only cause suicidal behavior inasmuch as they cause someone to conceptualize suicidal behavior as having a valued function in a given situation. Initial laboratory evidence is consistent with this valued function hypothesis [53].

## Conclusion

Biopsychosocial factor contributions to psychopathology are complex; failure to embrace this complexity results in low explanatory power. However, truly complex explanations are not comprehensible, limiting their ability to help us understand, predict, and prevent psychopathology. Psychological primitives can resolve this dilemma – they account for biopsychosocial complexity while maintaining comprehensibility (Table 1). The primitive-based approach does not eliminate a role for biopsychosocial factors in psychopathology; rather, it recasts these factors as indeterminate causal influences on psychological primitives (Fig. 1). This approach has generated major advances in affective science and should be equally applicable to psychopathology.

## Data Availability

Not applicable.

## Footnotes

1 The term ‘suicidality’ is used in this paper to refer to all suicide-related perceptions, cognitions, emotions, and behaviors. It is used instead of more specific terms because all discussions and conclusions are relevant to all suicide-related phenomena rather than just a few specific forms of suicidality (e.g., thoughts, attempts).

## References

[1] Cilliers P (2000). Rules and complex systems. Emergence..

[2] Poli R (2013). A note on the difference between complicated and complex social systems. Cadmus..

[3] Holland JH (1992). Complex adaptive systems. Daedalus..

[4] Miller JH, Page SE (2009). Complex adaptive systems: an introduction to computational models of social life.

[5] Edelman GM, Gally JA (2001). Degeneracy and complexity in biological systems. PNAS..

[6] Mason PH (2010). Degeneracy at multiple levels of complexity. Biol Theory.

[7] Mason PH, Winter B, Grignolio A (2015). Hidden in plain view: degeneracy in complex systems. Biosystems..

[8] Whitacre J, Bender A (2010). Degeneracy: a design principle for achieving robustness and evolvability. J Theor Biol.

[9] Franklin JC, Ribeiro JD, Fox KR (2017). Risk factors for suicidal thoughts and behaviors: a meta-analysis of 50 years of research. Psychol Bull.

[10] Huang X, Ribeiro JD, Franklin JC. The differences between suicide ideators and suicide attempters: simple, complicated, or complex? PsyArXiv. 2019. doi:10.31234/osf.io/8tuqg. 10.1037/ccp0000498 32105092

[11] Huang X, Rootes-Murdy K, Bastidas DM, Nee DE, Franklin JC. Brain abnormalities associated with self-injurious thoughts and behaviors: a meta-analysis of neuroimaging studies. bioRxiv. 2019. 10.1101/526525. 10.1038/s41598-020-59490-6 PMC7016138 32051490

[12] Walsh CG, Ribeiro JD, Franklin JC (2017). Predicting risk of suicide attempts over time through machine learning. Clin Psychol Sci.

[13] Walsh CG, Ribeiro JD, Franklin JC (2018). Predicting suicide attempts in adolescents with longitudinal clinical data and machine learning. J Child Psychol Psychiatry.

[14] Ribeiro Jessica D., Huang Xieyining, Fox Kathryn R., Walsh Colin G., Linthicum Kathryn P. (2019). Predicting Imminent Suicidal Thoughts and Nonfatal Attempts: The Role of Complexity. Clinical Psychological Science.

[15] Fox KR, Huang X, Guzman-Daireaux EM, Funsch K, Cha CB, Ribeiro JD, Franklin JC. How good are interventions of self-injurious thoughts and behaviors? A meta-analysis of 345 randomized controlled trials. In: The Annual Meeting of the Association for Behavioral and Cognitive Therapies. Nov 21–24, 2019, Atlanta, GA. https://eventscribe.com/2019/ABCT/fsPopup.asp?Mode=presInfo&PresentationID=603431

[16] Nock MK, Ramirez F, Rankin O (2019). Advancing our understanding of the who, when, and why of suicide risk. JAMA Psychiatry..

[17] Klonsky ED. The role of theory for understanding and preventing suicide (but not predicting it): a commentary on Hjelmeland and Knizek. Death Stud. 2019. 10.1080/07481187.2019.1594005. 10.1080/07481187.2019.1594005 30985259

[18] Van Orden KA, Witte TK, Cukrowicz KC, Braithwaite SR, Selby EA, Joiner TE (2010). The interpersonal theory of suicide. Psychol Rev.

[19] Winograd T (1978). On primitives, prototypes, and other semantic anomalies. Theoretical issues in natural language processing – 2.

[20] Barrett LF (2009). The future of psychology: connecting mind to brain. Perspect Psychol Sci.

[21] Lindquist KA (2013). Emotions emerge from more basic psychological ingredients: a modern psychological constructionist model. Emotion Rev.

[22] Barrett LF (2012). Emotions are real. Emotion..

[23] Barrett LF, Satpute AB (2013). Large-scale brain networks in affective and social neuroscience: towards an integrative functional architecture of the brain. Curr Opin Neurobiol.

[24] Ortony A, Turner TJ (1990). What's basic about basic emotions?. Psychol Rev.

[25] Russell JA (2003). Core affect and the psychological construction of emotion. Psychol Rev.

[26] Gendron M, Barrett LF (2009). Reconstructing the past: a century of ideas about emotion in psychology. Emotion Rev..

[27] Titchener EB (1898). The postulates of a structural psychology. Phil Rev.

[28] Wundt WM, Judd CH. Outlines of Psychology. 1897. Classics in the History of Psychology. https://psychclassics.yorku.ca/Wundt/Outlines/. Accessed 23 Sept 2019.

[29] Hoemann K, Barrett LF (2019). Concepts dissolve artificial boundaries in the study of emotion and cognition, uniting body, brain, and mind. Cognit Emotion.

[30] Lebois LAM, Wilson-Mendenhall CD, Simmons WK, Barrett LF, Barsalou LW. Learning situated emotions. Neuropsychologia. 2018. 10.1016/j.neuropsychologia.2018.01.008. 10.1016/j.neuropsychologia.2018.01.008 PMC6037607 29330097

[31] Lindquist KA, Barrett LF (2012). A functional architecture of the human brain: emerging insights from the science of emotion. Trends Cognit Sci.

[32] Lindquist KA, Wager TD, Kober H, Bliss-Moreau E, Barrett LF (2012). The brain basis of emotion: a meta-analytic review. Behav Brain Sci.

[33] Oosterwijk S, Lindquist KA, Anderson E, Dautoff R, Moriguchi Y, Barrett LF (2012). States of mind: emotions, body feelings, and thoughts share distributed neural networks. NeuroImage..

[34] Siegel EH, Sands MK, Van den Noortgate W (2018). Emotion fingerprints or emotion populations? A meta-analytic investigation of autonomic features of emotion categories. Psychol Bull.

[35] Barsalou LW (2009). Simulation, situated conceptualization, and prediction. Phil Trans Biol Sci.

[36] Barrett LF, Lindquist KA, Bliss-Moreau E, Duncan S, Gendron M, Mize J, Brennan L (2007). Of mice and men: natural kinds of emotions in the mammalian brain? A response to Panksepp an izard. Perspect Psychol Sci.

[37] Wilson-Mendenhall CD, Barrett LF, Barsalou LW (2013). Neural evidence that human emotions share core affective properties. Psychol Sci.

[38] Clark-Polner E, Johnson TD, Barrett LF (2016). Multivoxel pattern analysis does not provide evidence to support the existence of basic emotions. Cereb Cortex.

[39] Nosofsky RM, Pothos E, Willis A (2011). The generalized context model: an exemplar model of classification. Formal approaches in categorization.

[40] Gelman SA (2009). Learning from others: children’s construction of concepts. Ann Rev Psychol.

[41] Gendron M, Lindquist KA, Barsalou L, Barrett LF (2012). Emotion words shape emotion percepts. Emotion..

[42] Lindquist KA, Barrett LF (2008). Constructing emotion: the experience of fear as a conceptual act. Psychol Sci.

[43] Barrett LF, Adolphs R, Marsella S, Martinez A, Pollak SD (2019). Emotional expressions reconsidered: challenges to inferring emotion from human facial movements. Psychol Sci Public Interest.

[44] Gendron M, Roberson D, van der Vyver JM, Barrett LF (2014). Perceptions of emotion from facial expressions are not culturally universal: evidence from a remote culture. Emotion..

[45] Gendron M, Roberson D, van der Vyver JM, Barrett LF (2014). Cultural relativity in perceiving emotion from vocalizations. Psychol Sci.

[46] Lindquist KA, Gendron M, Barrett LF, Dickerson BC (2014). Emotion perception, but not affect perception, is impaired with semantic memory loss. Emotion..

[47] Touroutoglou A, Lindquist KA, Dickerson BC, Barrett LF (2015). Intrinsic connectivity in the human brain does not reveal networks for ‘basic’ emotions. Soc Cognit Affect Neurosci.

[48] Elsey JWB, Kindt M (2017). Breaking boundaries: optimizing reconsolidation-based interventions for strong and old memories. Learn Mem.

[49] Nock MK, Green JG, Hwang I, McLaughlin KA, Sampson NA, Zaslavsky AM, Kessler RC (2013). Prevalence, correlates, and treatment of lifetime suicidal behavior among adolescents: results from the National Comorbidity Survey Replication Adolescent Supplement. JAMA Psychiatry.

[50] Mishara BL (1999). Conceptions of death and suicide in children ages 6-12 and their implications for suicide prevention. Suicide Life Threat Behav.

[51] Pestian JP, Matykiewicz P, Linn-Gust M, South B, Uzuner O, Wiebe J, Cohen KB, Hurdle J, Brew C (2012). Sentiment analysis of suicide notes: a shared task. Biomed Inform Insights.

[52] Sendbuehler JM (1973). Attempted suicide: a description of the pre and post suicidal states. Can Psychiatr Assoc J.

[53] Franklin Joseph C., Huang Xieyining, Bastidas Diana (2019). Virtual reality suicide: Development of a translational approach for studying suicide causes. Behaviour Research and Therapy.

